# Is the early identification and referral of suspected head and neck cancers by community pharmacists feasible? A qualitative interview study exploring the views of patients in North East England

**DOI:** 10.1111/hex.13816

**Published:** 2023-07-17

**Authors:** Susan M. Bissett, Andrew Sturrock, Marco Carrozzo, Rachel Lish, Debora Howe, Susan Mountain, Michael Nugent, James O'Hara, Adam Todd, Scott Wilkes, Philip M. Preshaw

**Affiliations:** ^1^ School of Dental Sciences Newcastle University Newcastle upon Tyne UK; ^2^ Department of Nursing Midwifery and Health Northumbria University Newcastle upon Tyne UK; ^3^ Directorate of Multiprofessional Education, NHS England North East & Yorkshire Oral Health Improvement, Health Education England–North East North Cumbria Newcastle Upon Tyne UK; ^4^ Northern Head and Neck Cancer Charity (NHANCC) Northumberland UK; ^5^ Faculty of Health Sciences Patient/Public Involvement & Engagement Group, Sunderland University Sunderland UK; ^6^ Department of Oral and Maxillofacial Surgery Sunderland Royal Hospital Sunderland UK; ^7^ Ear, Nose and Throat Department Freeman Hospital Newcastle upon Tyne UK; ^8^ Population Health Sciences Institute Newcastle Univeristy Newcastle upon Tyne UK; ^9^ School of Pharmacy Newcastle University Newcastle upon Tyne UK; ^10^ School of Medicine University of Sunderland Sunderland UK; ^11^ School of Dentistry University of Dundee Dundee UK

**Keywords:** community pharmacy, early identification, head and neck cancer, pharmacists, qualitative, referral, screening

## Abstract

**Introduction:**

Head and neck cancer (HNC) is the eighth most common cancer in the United Kingdom. Survival rates improve when the cancer is diagnosed at an early stage, highlighting a key need to identify at‐risk patients. This study aimed to explore opportunistic HNC identification and referral by community pharmacists (CPs) using a symptom‐based risk assessment calculator, from the perspective of patients with a diagnosis of HNC.

**Methods:**

Purposive sampling was used to recruit patients from the HNC pathway in three large teaching hospitals in Northern England. Qualitative methodology was used to collect data through an iterative series of semistructured telephone interviews. Framework analysis was utilised to identify key themes.

**Results:**

Four main themes were constructed through the analytic process: (1) HNC presentation and seeking help; (2) the role of the CP; (3) public perception of HNC and (4) the role of a symptom‐based risk calculator. Participants agreed that CPs could play a role in the identification and referral of suspected HNCs, but there were concerns about access as patients frequently only encounter the medicine counter assistant when they visit the pharmacy. HNC symptoms are frequently attributed to common or minor conditions initially and therefore considered not urgent, leading to delays in seeking help. While there is public promotion for some cancers, there is little known about HNC. Early presentation of HNC can be quite variable, therefore raising awareness would help. The use of a symptom‐based risk calculator was considered beneficial if it enabled earlier referral and diagnosis. Participants suggested that it would also be useful if the public were made aware of it and could self‐assess their symptoms.

**Conclusion:**

In principle, CPs could play a role in the identification and referral of HNC, but there was uncertainty as to how the intervention would work. Future research is needed to develop an intervention that would facilitate earlier identification and referral of HNC while not disrupting CP work and that would promote HNC and the risk calculator more widely.

**Patient or Public Contribution:**

Patient and public involvement and engagement (PPIE) was integrated throughout the project. Initially, the proposal was discussed during a Cancer Head and Neck Group Experience (CHANGE) PPIE meeting. CHANGE was set up to support HNC research in 2018. The group is composed of seven members (four female, three male) with an age range of 50–71 years, who were diagnosed at Sunderland Royal Hospital. A patient representative from the University of Sunderland PPIE group and a trustee of the Northern HNC Charity were recruited as co‐applicants. They attended project management group meetings and reviewed patient‐facing documentation.

## 1 INTRODUCTION

Head and neck cancer (HNC) is the eighth most common cancer in the United Kingdom and incidence rates are continuing to rise. HNC includes cancers of the oral cavity, larynx, pharynx, nose, throat, tonsils and salivary glands. HNC accounts for 3% of all new cancers diagnosed in the United Kingdom.^1^ The North East of England has been identified as the region with the highest incidence of HNC,^2^ with evidence supporting both increased incidence and mortality rates in areas of high deprivation.^3^ Avoidable premature mortality amongst cancer patients is higher in the United Kingdom compared with the mean survival in Europe, and earlier detection could eradicate the gap.^4^, ^5^ A key ambition of the 2019 NHS Long Term Plan is to improve early‐stage cancer diagnosis, with a target of 75% of patients diagnosed early by 2028.^6^ However, it is recognised that at the point of diagnosis, many patients with HNC have progressed to advanced disease status^7^, ^8^; whereas survival rates improve when the cancer is diagnosed at an early stage, highlighting a key need to identify at‐risk patients.^9^

HNC is mostly diagnosed upon symptomatic presentation, which varies amongst patients. While oral cancers are frequently preceded by potentially malignant oral disorders (e.g., leukoplakia, erythroplakia, oral lichen planus etc.), other HNCs may initially present with ear pain, a sore throat or a neck obstruction. This means that patients may present their symptoms in a variety of healthcare settings.^10^ Community pharmacies are easily accessible, with 90% of the UK population living within 20 minutes of their local pharmacy.^11^ The 2022 Pharmacy Advice Audit report confirmed that UK pharmacies are saving 32 million general practitioner (GP) appointments per year. These data are being used to advocate for walk‐in pharmacy advice services.^12^ In June 2022, NHS England announced that pilot schemes would be created to allow community pharmacists (CPs) to refer potential cancer cases directly to hospitals.^13^ In addition to the routine provision of over‐the‐counter treatments and advice for conditions that may be the result of an undetected HNC (e.g., persistent oral ulceration), CPs participate in healthy living promotion campaigns each year (e.g., smoking cessation counselling).^14^ Research conducted alongside this study explored the views of CPs regarding their involvement in the early identification and referral of HNCs and found that while they would support such an intervention, further work was needed to develop a sustainable and cost‐effective intervention that would include CP training for optimum patient care.^15^

Clinical decision‐making tools and risk calculators are available for a number of common cancers and are routinely used to aid prompt referral of high‐risk individuals to specialist clinics for further assessment.^16^, ^17^, ^18^ A validated HNC symptom‐based risk assessment (www.ORLhealth.com) has been produced, which provides a straightforward web‐based tool, that could potentially be used in a pharmacy setting.^19^ Accordingly, our study aim was to explore opportunistic HNC identification and referral in a community pharmacy setting through the perspectives of patients with a diagnosis and lived experience of HNC.

## 2 METHODS

To enhance the reporting of this study, the COnsolidated criteria for REporting Qualitative studies checklist^20^ was used (see Supporting Information: File 1).

### 2.1 Study design

Data were collected through an iterative series of semistructured interviews with patients who had a diagnosis of HNC. An initial topic guide was developed by the lead investigators (S. M. B. and A. S.) based around the following criteria: awareness of HNC symptoms; use of risk prediction tools and perceptions of potential future roles for CPs in HNC identification/referral. The semistructured style of interview provided flexibility to explore other topics that arose.

### 2.2 Identification, invitation and recruitment of participants

Purposive sampling was used with the patients being invited to be contacted by the researchers via their HNC care team and hospital consultants working in three large teaching hospitals in Newcastle upon Tyne and Sunderland, United Kingdom. Patients were contacted by telephone, informed about the study and invited to participate by the lead investigator (S. M. B.), who also conducted the interviews. The overall objectives of the study, information about the funding source and key points relating to participation, confidentiality and anonymisation of data transcripts were explained to the participants and informed consent was obtained before commencing the interviews.

### 2.3 Data collection and analysis

Individual semistructured interviews lasting up to 60 minutes were undertaken by telephone with the participant at home, sometimes alone and other times accompanied by a relative. Field notes were collated during the interview. Interviews were audio‐recorded and transcribed verbatim to facilitate analysis. A conversational style of interviewing was used to encourage a comfortable and fluent dialogue. An iterative cycle of data collection and analysis facilitated the adaption of the topic guide to enable further exploration of new lines of enquiry in subsequent interviews. In the absence of a priori theory, framework analysis was utilised to identify key themes.^21^ Initial analysis allowed familiarisation with the data, and this was followed by a process of revisiting data via the transcripts alongside audio‐recording with manual coding of concepts to develop a thematic framework. There were no repeat interviews. Themes were reviewed by the lead investigators (S. M. B. and A.S.) and discussed with the wider research and patient, public, involvement and engagement team to establish definitive concepts.

### 2.4 Reflexivity statement

The research team included experts in oral medicine, HNC, pharmacy, periodontology and general medicine. S. M. B. obtained informed consent and interviewed the participants. S. M. B. is a female researcher with a background in dental hygiene and experience in oral and dental research using qualitative and mixed methods. S. M. B. had not met the participants before, but they were informed that she worked at the Dental School.

## 3 RESULTS

### 3.1 PARTICIPANTS' CHARACTERISTICS

Nineteen patients were approached: and 6 were lost to follow up or did not want to take part, whereas 13 participants signed consent and completed an interview (see Table 1). They were eight males and five females, aged between 42 and 79 years old. They were recruited from HNC pathways in three large hospitals in North East England: six from the Department of Oral and Maxillofacial Surgery, Sunderland Royal Hospital, four from Ear, Nose and Throat Department, Newcastle Freeman Hospital and three from Oral Medicine Department, Newcastle Dental Hospital. All participants received a referral for their symptoms from their primary care GP or general dental practitioner (GDP). Eight participants were ex‐smokers and three had never smoked. Ten reported drinking alcohol within the current government guidelines and three admitted to drinking more than the recommended limits. One participant received a positive human papillomavirus diagnosis following his HNC diagnosis. Seven were ‘problem‐orientated dental attenders’ or irregular attenders at a dentist, only seeking care when having dental pain and problems, rather than attending for regular preventative care; and six were reluctant to go to the doctor, only going if they had to. Seven said they had previously attended their pharmacy for advice, while six said they went for prescription dispensing only.

**Table 1:** Participants' characteristics.

ID	Completed Interview	Gender	Age	Hospital	Employment	Smoking history	Alcohol history	Dental care attendance	Medical care attendance	Pharmacy attendance
SR01	Yes	M	53	SRCDC	Unemployed	Nonsmoker	<government guidelines	POA	POA	PO
FH01	Yes	F	42	NFHENT	Charity work as a coach	Ex‐smoker	<government guidelines	RA	POA	PAS
FH02	Yes	M	64	NFHENT	Retired	Ex‐smoker	>government guidelines	POA	POA	PAS
DH01	Yes	M	73	NDHOS	Retired	Nonsmoker	<government guidelines	RA	POA	PAS
DH03	Yes	M	79	NDHOS	Retired	Nonsmoker	<government guidelines	POA	POA	PO
DH02	Yes	F	56	NDHOS	Pharmacy (at counter)	Ex‐smoker	<government guidelines	RA	RA	PAS
SR03	Yes	F	55	SRCDC	Accountant	Nonsmoker	<government guidelines	RA	RA	PAS
FH05	Yes	M	74	NFHENT	Retired	Ex‐smoker	>government guidelines	POA	POA	PAS
SR04	Yes	M	60	SRCDC	Public transport	Nonsmoker	<government guidelines	RA	RA	PO
SR05	Yes	M	60	SRCDC	Window supplier	Ex‐smoker	<government guidelines	RA	RA	PO
FH06	Yes	F	61	NFHENT	Care assistant	Ex‐smoker	<government guidelines	RA	RA	PAS
SR09	Yes	M	59	SRCDC	Engineer	Ex‐smoker	>government guidelines	POA	RA	PO
SR08	Yes	F	61	SRCDC	Retired	Ex‐smoker	<government guidelines	POA	RA	PO

Abbreviations: F, female; M, male; NDHOS, Newcastle Dental Hospital Oral Surgery department; NFHENT, Newcastle Freeman Hospital Ear, Nose and Throat department; PAS, prescription, advice, screening; PO, prescription only; POA, problem‐orientated attendance; RA, regular attendance; SRCDC, Sunderland Royal Cancer Diagnostic Centre; <, less than; >, more than.

### 3.2 Findings

The thematic analysis methodology revealed four main themes that were identified through the analytic process.

#### 3.2.1 HNC presentation and seeking help

The participants had diagnoses of HNC that included cancers of the lip, tonsils, tongue, vocal cords and throat. The initial sign or symptom was often innocuous and included a persistent ulcer or sore, a lump or swelling, earache or hoarseness of the voice; and there was frequently no pain. Participants described initially attributing the signs and symptoms to a minor ailment like a cold sore; or an abrasion resulting from a broken tooth, or a side‐effect of medication being taken for another chronic condition or a vitamin deficiency. Some participants used a variety of ways to self‐treat their symptoms, but as time passed and symptoms persisted or worsened, participants would start to consider seeking help, although sometimes it took a family member or friend to persuade them to seek advice. Where there was inertia, this was attributed to living alone (easier to ignore), not wanting to waste anyone's time, a phobia or not considering it an emergency.

Well, it started about, just after Christmas a couple of years back when my, my voice started getting very croaky. And I let it go for a couple of months, and when a friend of mine actually said, you know, I should get this, get it checked out really. And, because it wasn't getting any better, and my voice was getting increasingly croaky. So that's the reason I went to the doctors, it was to get it, you know, to see why my voice was croaky.… And there was no pain at any time. [patient with throat cancer, FH05]

Once participants had made the decision to get help, some participants found it straightforward to access care and get an immediate referral, whereas others described difficulty in being seen and delays in getting a diagnosis. Some experiences were exacerbated by the COVID‐19 pandemic, either due to not having access to face‐to‐face appointments or having to cope on their own due to ‘attend‐alone’ protocols.

I noticed, well my husband noticed a lump in my neck in April 2020 and so it was the very start of lockdown. And then I rang the GP who said ‘well, Mrs [name], you're at far greater risk of COVID than you are of cancer’. So, I rang them back in a few weeks and I said it's still here and I'm worried, I'm having pains down my arm, and he said ‘well, what are you worried about?’ and I said ‘well, I'm worried that I might have cancer or I'm going to have a heart attack’ and they laughed. They said ‘no, Mrs [name], that's not going to happen. If you're still concerned in a few more weeks let me know because under the current circumstances, it wouldn't be wise to make any referrals’. It took until August of me ringing and they, they eventually sent me for a scan on my neck and within three days it came back, and they still didn't see me. They rang me and said, ‘I can't tell you that it's cancer over the phone, but we need you to go to the hospital’, and that's when it was all explained to me. [patient with cancer of the tonsil, FH01]

#### 3.2.2 Role of the CP

All participants agreed that CPs were highly qualified and could play a role in the early referral of HNC, particularly with additional training. The pandemic had shown that CPs could undertake extended roles, such as in delivering COVID‐19 and influenza vaccinations. CPs were often attached to a medical centre or on the ‘high street' with extended opening hours and no appointment was necessary. There were consultation spaces created in many pharmacies, that provided an option for privacy; and, furthermore, as a face‐to‐face appointment with a GP/GDP was often difficult to obtain following the COVID‐19 pandemic, CPs were considered much more accessible than GPs.

…if there'd been a pharmacist and I'd went there, they might have turned round and said, ‘That isn't off tablets, tablets don't do that’. But I didn't know any different, you know… With training I think they would, yes, they would be the right people. You know a pharmacist's role at the minute is a different thing, isn't it? We've seen it with the pandemic, I mean they're giving flu injections, they were doing the virus, you know inoculations… Well, being honest I think there's more pharmacists than what there is doctors… So you know, surely they can come up and go, ‘Mr [name] like we'd better refer you back to the doctors’. [patient with cancer of the vocal chords, FH02]

Notwithstanding, there was a perception that CPs' main occupation was dispensing medicines. If they were to offer advice, it was in relation to a specific pharmaceutical regimen; or they discussed potential side effects or suggested over the counter, possibly cheaper alternatives to the prescribed medicine. CPs were also regarded as being incredibly busy, frequently observed with a queue of people waiting. Often, the CP would spend much of their time ‘hidden around the back’ with possibly very little time to spare for consultations. In addition, the counter space was not considered an appropriate place for engaging in confidential and potentially highly personal forms of health‐related discourse. Even with a consultation room, it was considered off‐putting that to access the CP, there may still have to be a conversation with the medicine counter assistant first. Finally, consideration was given to the seriousness of the potential diagnosis and prognosis. Would the consultation be managed appropriately, and would the CP be able to offer support and compassion if the person became distressed?

A pharmacist could definitely offer that service, I would've said. But I don't know if it would be a port of call that I would consider, if I'm honest with you. I wouldn't consider walking into the pharmacist down here. I live in XXX [a village in a large county of North East England]. I wouldn't consider walking into that Centre, that girl behind the counter, chap behind the counter. Would you mind having a look at this? I don't know if that's a good thing or a bad thing, but that's truth of it…[I] was spitting blood…I was in a pretty dark place at the time.…and, and that's something you don't want to hear in the pharmacist, as well. It surely doesn't look quite good. [patient with throat cancer, SR05]

#### 3.2.3 Public perception of HNC—what does HNC look like?

HNC is a category of cancer that manifests in a diverse way due to the anatomy of the head and neck. Participants described being unaware of HNC and not knowing what to look out for. Notwithstanding the lack of awareness or ‘silence’ surrounding HNC, even a small tumour that is caught early, can have a hugely distressing impact on the person's life and rehabilitation. This quote is from a patient who considered their cancer to be a ‘minor’ cancer, as they didn't realise how serious it was.

And I just think to myself, I see the advertisements on television. And you see people with breast cancer, lung cancer, and I think I've got nothing and then the nurses [told me] it's cancer, [and] cancer is something. But I find it hard to think that it's serious you know…this is probably one of the problems when I've seen the picture on the internet. I certainly thought, oh, I've got that. But I still didn't do nothing about it because I didn't think it was that serious… But like I said, I also didn't know, which I know now, that I'm under super…, like under consultation now for the next five years. So I mean, that's a big thing in your life. They said the first two years it can come back. And I said, oh comes back on me lips? He says no it can comeback anywhere. I says, well, I never knew that, nobody taught me that. [patient with cancer of the lip, SR01]

Some of the participants questioned why some cancers receive more healthcare promotion than others. Breast cancer, for example, was mentioned frequently, being publicised in campaigns that were advertised ‘everywhere’, with self‐examination recommended, and it was clear what to look out for. While raising awareness about HNC was felt to be a good idea, the fact that HNC presents in multiple ways made it more complex. Nevertheless, using posters, adverts via multiple sources of media and personal testimonials was felt to be important. In the next quote, a participant describes how their cancer was found and how their cancer journey was featured on the Macmillan website.

Like I say…I'm only telling you what's happened to me. Mine was basically like I said; it wasn't something I was having trouble with. I didn't have a sore throat; I got two teeth taken out [which was unrelated but led to the diagnosis]. And, touch wood, from that, like I say, I wouldn't say it was a ‘run of the mill, I'm suffering from a sore throat, I had a hoarse voice’, or anything like that. Mine was totally different [no symptoms], but anybody who's had teeth taken out, found a lump anywhere, go to the doctor straight away you know, get it checked out. Yeah, I did a story on the Macmillan website…March 2020; you'll see my story and me wife's story. [patient with cancer of the tonsil, SR04]

#### 3.2.4 Role of a symptom‐based risk calculator

During the interviews the use of the online symptom‐based risk calculator for HNC, http://orlhealth.com/ was discussed. The participants were told that the calculator consists of 14 questions related to symptoms of HNC such as dysphagia, hoarseness, ulcer, neck mass, persistent skin lesion, sensation of a lump in the throat, otalgia and odynophagia. Participants were in agreement that anything that would enable a faster referral would be good. CPs were known to be highly qualified professionals, but also very busy, and as the calculator was a simple tool, it was suggested that medicine counter assistants in the pharmacy could use it. Furthermore, some thought it would be good if it was accessible to the public.

Absolutely, couldn't agree more. Had I had something like that, I'm sure we could have got that diagnosis a little bit quicker. You know, maybe a couple of months quicker, which makes the world of difference when you've got something like that. And I absolutely think had there been a tool like that, even if it was online or whatever, I would've used that without a shadow of a doubt. Absolutely, one hundred percent, one hundred percent. [patient with cancer of the tonsil, SR05]

In addition to questioning the practicalities of completing an HNC risk consultation by a CP, such as time constraints and the need for privacy, some participants wondered how the conversation would be initiated. As medicine counter assistants and CPs are frequently giving advice, would they know when to suggest a risk calculator? Would it be up to the customer to ask for a risk assessment?

If the pharmacist asked me a few questions would I have waited about for them to do it? Probably not. I would have tended to go to my GP late at night, after work and that. Is it a good idea? Yes, I definitely think it is because there will be some people that are not like me who would be happy to wait. But who would ask all those questions because when I go to the pharmacy it tends to be effectively shop assistants that help me with my prescription. Would they have the sense to say, ‘Oh, [name] has been in three times and had Difflam or had Bonjella’.…Would it rely on me saying, ‘I've had this ulcer for three weeks, is there anything I can use on it?’ At that point they'd say to the pharmacist, ‘This lady…’ or would they just turn round and say, ‘Try Bonjella, or rinse it with salt water’.…I think for me I would want to see a poster in the pharmacy saying, ‘Have you had a sore mouth for more than three weeks? Have you had a sore throat for more than three weeks? If so, talk to your pharmacist and they may be able to suggest something’. Or ‘If so, look up this [the symptom based risk calculator]’. [patient with tongue cancer, SR03]

## 4 DISCUSSION

This qualitative semistructured interview study explored the views of patients regarding the feasibility of CPs providing a role in the early identification and referral of suspected HNC. The participants shared a diagnosis of HNC, but they were in different stages of their HNC pathway with some having had surgery recently and others being some way into remission. They all agreed that anything that reduces the length of time it takes to diagnose HNC is valued as it will improve outcomes. During the interviews, an intervention to evaluate risk and facilitate early referral was unpacked in practical terms.

Regarding the role of the CP, patients perceived that while CPs were highly trained in medicines and no appointment was needed to see them, there were barriers to access as CPs were incredibly busy and medicine counter assistants were the first person, and sometimes the only person, the patients spoke to. This raised the question of whether the medicine counter assistant could have a role in the early identification of suspected HNC. This idea was further strengthened if the consultation included the use of the symptom‐based risk calculator, as this was designed to be quick and simple to complete and didn't require any expertise. Furthermore, as CPs tend to rely on indirect referrals,^15^ or signposting, it would not make a difference who delivered the intervention if the ultimate advice was for the patient to go to their GP or GDP. The Northern Cancer Alliance has a website with an urgent referral form, but it is currently only available for GPs or GDPs to complete.^22^ Future research would be needed to explore the role of a medicine counter assistant in delivering this intervention, in addition to investigating the possibilities for adapting the urgent referral form for nondentists/medics to complete.

Awareness of HNC was poor and this was considered to have an impact on health seeking behaviour. Patients described the varied and sometimes innocuous symptoms of HNC and how the initial presentation of, for example, an ulcer or a croaky voice did not seem to indicate an emergency or something life threatening. Research has found that those cancers that present with vague, ‘non‐urgent’ symptoms can take a median of 34 days longer to diagnose.^23^ There was uncertainty as to why certain cancers and not others were the subject of healthcare promotion. If people were given access to information in adverts, campaigns or in the media, participants felt it would alert them to the fact that an ulcer or croaky voice, for example, could be HNC. Raising awareness seemed to be important as the location of HNC tumours often resulted in treatment that was highly invasive and traumatic. Indeed, HNC survivors often suffer with ongoing alteration of daily functions, psychological distress and ongoing issues with speech and swallowing.^24^, ^25^, ^26^ It was considered that perhaps a pharmacy could be less formal and therefore a preferred environment to ask for advice regarding these symptoms; but there were also concerns about the lack of privacy and whether the CP would recognise the potential risk of HNC when the early symptoms can be so common and minor.

The data suggested that the idea of using a symptom‐based risk calculator was good if it would help earlier identification or referral, although research is yet to identify if clinical decision‐making tools are disruptive to consultation times, increasing them to an inappropriate degree.^27^ Furthermore, the calculator does not include premalignant oral conditions such as leukoplakia, erythroplakia and oral lichen planus. Notwithstanding, for cancer symptoms such as an ulcer or a croaky voice, the calculator would result in the recommendation of an urgent referral if the symptoms were persistent and unexplained, even with an absence of a history of smoking or excess alcohol consumption. It was suggested that if CPs were trained in the risk assessment tool and the symptoms and concept of ‘persistence’, this could improve the chances of earlier referral or signposting. Persistence is difficult to define for some symptoms of HNC, such as sore throat, but Tikka et al. suggest over 3 weeks for oral ulcer or swelling, dysphagia and under 3 weeks for a recent unexplained neck lump.^28^ Furthermore, there was some suggestion that if the risk calculator was more publicly advertised and available, it may help people to contact a healthcare professional earlier. The calculator also has functionality for printing the results, which the patient could then take to their GP/GDP appointment.

The promotion of HNC was considered important by all participants, who had poor awareness, indicating a need for more publicity. The United Kingdom has nominated 23rd September as HNC Awareness Day. In addition to the Northern Head and Neck Cancer Charity, which has supported this research, there are other cancer charities offering a range of resources.^29^ The Macmillan cancer support charity has information on all cancers, including HNC, and a function to chat to a specialist online; the Northern Cancer Alliance has information on HNC and a GP/GDP urgent referral function and the Mouth Cancer Foundation has an oral cancer self‐check video and testimonials.^22^, ^30^, ^31^, ^32^, ^33^ Furthermore, in January 2023, *The Times* published an article by the Scottish Health Correspondent, Helen Puttick, on the merits of the symptom‐based risk calculator.^34^ The participants in this study appeared to be largely unaware of these resources.

A strength of this study is that it is the first that we are aware of that explores the views of patients regarding a CP's role in the early detection and referral of HNC. Furthermore, it includes a variety of subsites and patients at different stages of the HNC pathway. A limitation is the potential for selection bias. Potential participants were identified and initially approached by their consultant. All participants had received a diagnosis of HNC as we wished to explore the views of those who had lived experience, and we recruited from three hospitals in the region to find a range of perspectives. Notwithstanding, our selection strategy may have influenced the findings, and this may limit their transferability to other areas of the country or to those presenting in emergency settings.

While the data support the concept of CPs delivering an intervention that could lead to earlier identification and referral of HNCs in principle, there was uncertainty as to how this would work in practice. Research conducted alongside this study explored the views of CPs regarding their involvement in such an intervention and although it was felt to be possible to support HNC awareness initiatives, early identification and referral, the findings suggested that further work was needed to develop a sustainable and cost‐effective intervention.^15^ This study found that regarding accessibility, there were concerns about waiting times, privacy, CP training and support, which would suggest the need to raise public awareness about the clinical role of a CP. CP capacity to deliver the intervention would also need to be assessed and reviewed locally to consider staff time and strategies to mitigate disruption. Furthermore, if a CP could make a 2‐week urgent referral via the Northern Cancer Alliance website, this could improve and add value to the intervention.

## 5 CONCLUSION

The findings of this study suggest that the principle of utilising CPs in the early identification and referral of suspected HNC was accepted by all participants, with the agreement that it may encourage some people to make an appointment with their GP/GDP. However, there was uncertainty as to how the intervention would work. Future research will need to look at intervention development and implementation that includes CP and the wider community pharmacy team training and capacity building; and the design and delivery of an effective HNC public health campaign.

## AUTHOR CONTRIBUTIONS

All authors contributed to the study conception, design, analysis and interpretation of data, and reviewed/revised the submission. Marco Carozzo, Michael Nugent and James O'Hara identified the patient participants. Susan M. Bissett took consent, acquired the data and drafted/reviewed the final article.

## CONFLICT OF INTEREST STATEMENT

The authors declare no conflict of interest.

## ETHICS STATEMENT

The study was reviewed and approved by North‐West Preston Research Ethics Committee (reference: 21/NW/0126) on 26.05.2021; and the HRA and Health and Care Research Wales, on 27.05.2021.

## Supporting information

Supporting information.

## Data Availability

The data that support the findings of this study are available from the corresponding author upon reasonable request.
